# Cost-effectiveness of massively parallel sequencing for diagnosis of paediatric muscle diseases

**DOI:** 10.1038/s41525-017-0006-7

**Published:** 2017-03-03

**Authors:** Deborah Schofield, Khurshid Alam, Lyndal Douglas, Rupendra Shrestha, Daniel G. MacArthur, Mark Davis, Nigel G. Laing, Nigel F. Clarke, Joshua Burns, Sandra T. Cooper, Kathryn N. North, Sarah A. Sandaradura, Gina L. O’Grady

**Affiliations:** 1 Faculty of Pharmacy, University of Sydney, Sydney, NSW Australia; 2 Murdoch Childrens Research Institute, Melbourne, VIC Australia; 3 Garvan Institute for Medical Research, Darlinghurst, NSW Australia; 4 Faculty of Medicine, Department of Paediatrics, University of Melbourne, Melbourne, VIC Australia; 5 Department of Clinical Genetics, Children’s Hospital at Westmead, Locked Bag 4001, Sydney, NSW Australia; 6 Institute for Neuroscience and Muscle Research, Kids Research Institute, Children’s Hospital at Westmead, Sydney, NSW Australia; 7 Analytic and Translational Genetics Unit, Massachusetts General Hospital, Boston, MA USA; 8 Program in Medical and Population Genetics, Broad Institute of Harvard and MIT, Cambridge, MA USA; 9 Department of Diagnostic Genomics, PathWest Laboratory Medicine, QEII Medical Centre, Nedlands, WA Australia; 10 Centre for Medical Research University of Western Australia, Harry Perkins Institute of Medical Research, Nedlands, WA Australia; 11 Faculty of Medicine, Discipline of Paediatrics and Child Health, University of Sydney, Sydney, NSW Australia; 12 Sydney Children’s Hospitals Network (Randwick and Westmead), University of Sydney, Sydney, NSW Australia; 13 Paediatric Neuroservices, Starship Children’s Health, Auckland, New Zealand

## Abstract

Childhood-onset muscle disorders are genetically heterogeneous. Diagnostic workup has traditionally included muscle biopsy, protein-based studies of muscle specimens, and candidate gene sequencing. High throughput or massively parallel sequencing is transforming the approach to diagnosis of rare diseases; however, evidence for cost-effectiveness is lacking. Patients presenting with suspected congenital muscular dystrophy or nemaline myopathy were ascertained over a 15-year period. Patients were investigated using traditional diagnostic approaches. Undiagnosed patients were investigated using either massively parallel sequencing of a panel of neuromuscular disease genes panel, or whole exome sequencing. Cost data were collected for all diagnostic investigations. The diagnostic yield and cost effectiveness of a molecular approach to diagnosis, prior to muscle biopsy, were compared with the traditional approach. Fifty-six patients were analysed. Compared with the traditional invasive muscle biopsy approach, both the neuromuscular disease panel and whole exome sequencing had significantly increased diagnostic yields (from 46 to 75% for the neuromuscular disease panel, and 79% for whole exome sequencing), and reduced the cost per diagnosis from USD$16,495 (95% CI: $12,413–$22,994) to USD$3706 (95% CI: $3086–$4453) for the neuromuscular disease panel and USD$5646 (95% CI: $4501–$7078) for whole exome sequencing. The neuromuscular disease panel was the most cost-effective, saving USD$17,075 (95% CI: $10,654–$30,064) per additional diagnosis, over the traditional diagnostic pathway. Whole exome sequencing saved USD$10,024 (95% CI: $5795–$17,135) per additional diagnosis. This study demonstrates the cost-effectiveness of investigation using massively parallel sequencing technologies in paediatric muscle disease. The findings emphasise the value of implementing these technologies in clinical practice, with particular application for diagnosis of Mendelian diseases, and provide evidence crucial for government subsidy and equitable access.

## Introduction

Childhood-onset muscle disorders present with hypotonia and delay of gross motor milestones. Congenital muscular dystrophies (CMD) are characterised by dystrophic features on skeletal muscle biopsy and commonly by elevated creatine kinase (CK) levels in blood. The (non-dystrophic) congenital myopathies, of which nemaline myopathy (NM) is a subtype, are characterised by specific morphological features on muscle biopsy. Massively parallel sequencing technology is transforming the diagnostic evaluation of this heterogeneous group of disorders.

Increasing evidence supports improved diagnostic success using both neuromuscular gene panels and whole exome sequencing (WES), with diagnosis rates ranging from 49 to 83%.^1–4^ However, there is limited evidence regarding the relative cost-effectiveness of diagnostic evaluation using massively parallel sequencing technology, compared with traditional diagnostic work-up using muscle biopsy, histological and biochemical analyses of muscle specimens, followed by Sanger sequencing of candidate genes. With genomic technologies rapidly entering clinical practice, evidence for cost effectiveness is crucial in many countries to obtain government subsidy for this new technology and thus ensure equity of access.

We have retrospectively evaluated the diagnostic outcomes for a cohort of 56 patients with childhood-onset muscle diseases (Supplementary Table e1) comparing traditional diagnostic techniques with massively parallel sequencing technologies, to evaluate the economic impact of a shift toward molecular-based diagnostics.

## Results

### Patient cohort and diagnostic outcomes

We identified a cohort of 58 patients (40 CMD patients and 18 NM patients), ascertained over 15 years from a publically funded, tertiary pediatric Australian neuromuscular centre. Demographic characteristics of the cohort are characterized in Table 1, and genetic diagnoses in Supplementary Table e1. Fifty-two patients were the index case and six were affected siblings. The mean duration of the diagnostic odyssey was 7.7 years (range 2 months to 26 years). Two CMD patients did not consent to further genetic evaluation and were excluded from further analysis. Thus, 56 patients were included in the final cohort.

**Table 1:** Demographic characteristics of the cohort

Characteristics	Number (%)
*Sex*	
Male	30 (53.6%)
Female	26 (46.4%)
*Clinical diagnosis*	
Congenital muscular dystrophies (CMD)	38 (67.9%)
Nemaline myopathy (NM)	18 (32.1%)
Parental consanguinity	7 (12.5%)
*Age at onset*	
Birth	34 (60.7%)
1^st^ Year	11 (19.6%)
2^nd^ Year	8 (14.3%)
>2^nd^ Year	3 (5.4%)

**Fig. 1:**
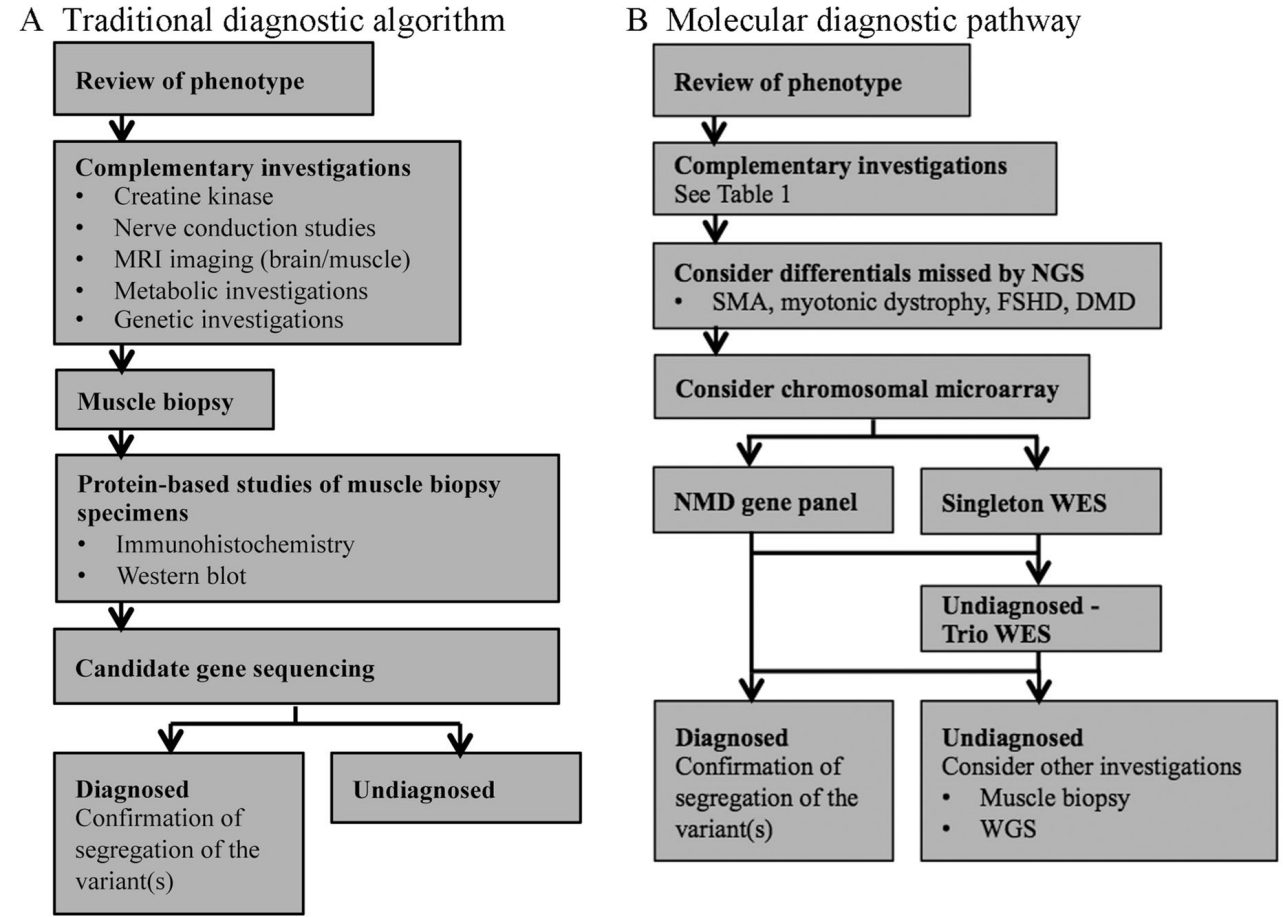
Proposed diagnostic algorithms. **a** shows the traditional diagnostic algorithm based on muscle biopsy and protein-based studies of muscle biopsy specimens, followed by candidate gene sequencing. Complementary investigations were selected in the cohort by treating clinicians based on the presenting phenotype. **b** shows the proposed molecular approach to diagnosis. Complementary investigations were included dependent on the presenting phenotype, based on Table 2. Molecular investigation using either a NMD gene panel or WES was performed prior to muscle biopsy. *NMD* neuromuscular disease, *WES* whole exome sequencing, *WGS* whole genome sequencing, *SMA* spinal muscular atrophy, *FSHD* facioscapular humeral dystrophy, *DMD* Duchenne muscular dystrophy

**Fig. 2:**
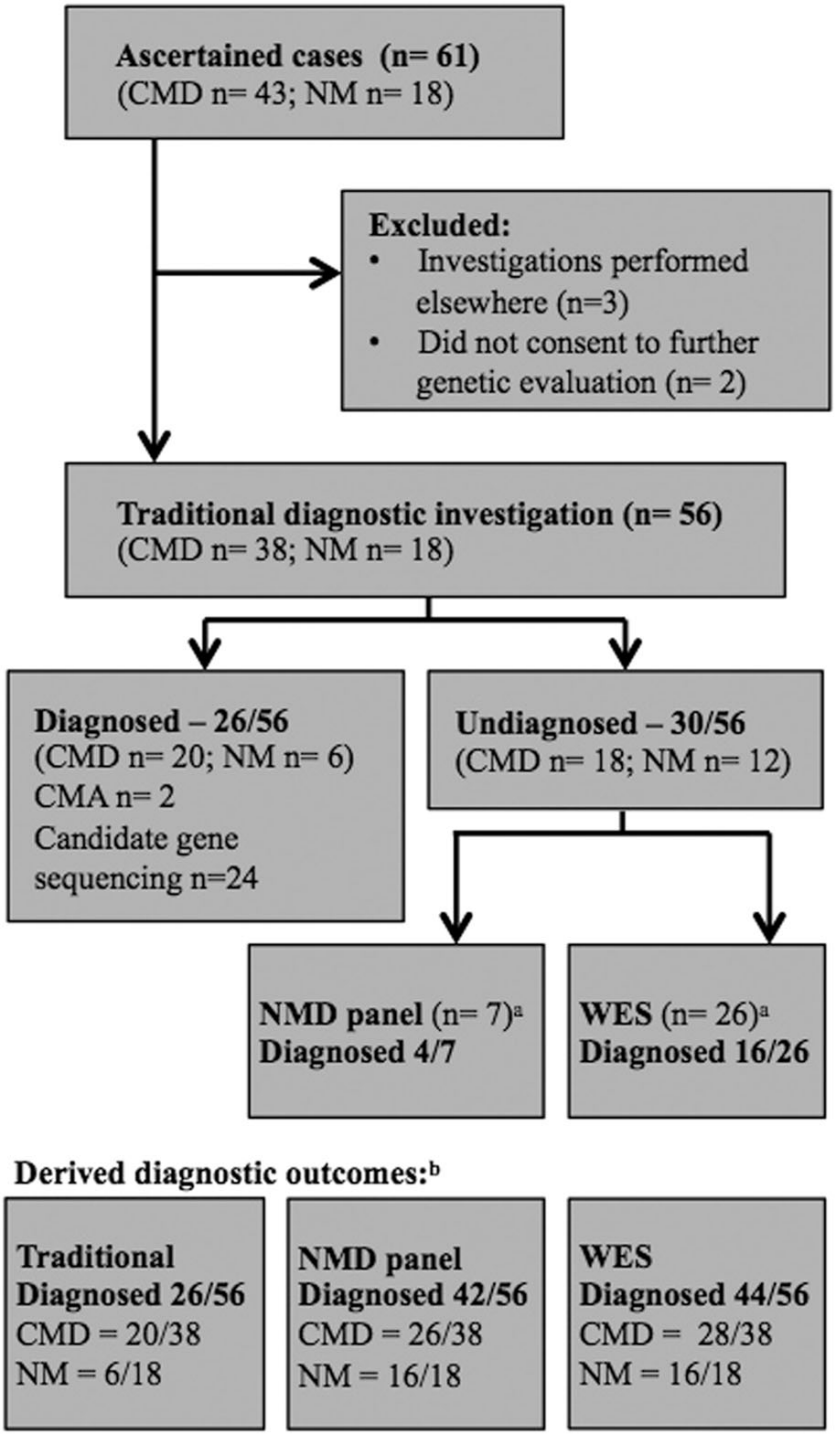
Case ascertainment and investigation. Ascertainment and diagnostic outcomes for the combined CMD and NM cohorts. **a** Three patients were investigated using only the NMD panel, four patients had both NMD panel and WES, and one sibling had neither NMD panel or WES while investigations were performed on the proband. **b** Diagnostic outcomes for the NMD panel are derived from data obtained from WES and based on previous studies comparing the diagnostic efficacy of WES and NMD panel testing, given only a small subset (*n* = 4) were investigated using both WES and the NMD panel. An assumption is made that patients diagnosed using candidate gene sequencing would also be diagnosed using CMA plus NMD panel or WES. *CMD* congenital muscular dystrophy, *CMA* chromosomal microarray, *NM* nemaline myopathy, *NGS* next-generation sequencing, *NMD* neuromuscular disease, *WES* whole exome sequencing

Using traditional diagnostic approaches (Fig. 1), a genetic diagnosis was achieved for 46% of patients (26/56), including two patients diagnosed by chromosomal microarray. The 30 patients who remained undiagnosed after traditional diagnostic investigations were investigated using WES, with the exception of three patients who had been investigated by the neuromuscular disease (NMD) panel without WES, and one sibling who had neither WES nor panel, while WES was performed on the proband (Fig. 2). This resulted in 18 additional diagnoses, including four patients with variants in newly identified disease genes: two CMD siblings contributed to identification of *PIGY* ^5^ and two siblings with NM contributed to identification of *LMOD3*.^6^ Two patients had variants in recently published genes (*MICU1* and *GFPT1*).

### Cost-effectiveness from economic analysis

**Fig. 3:**
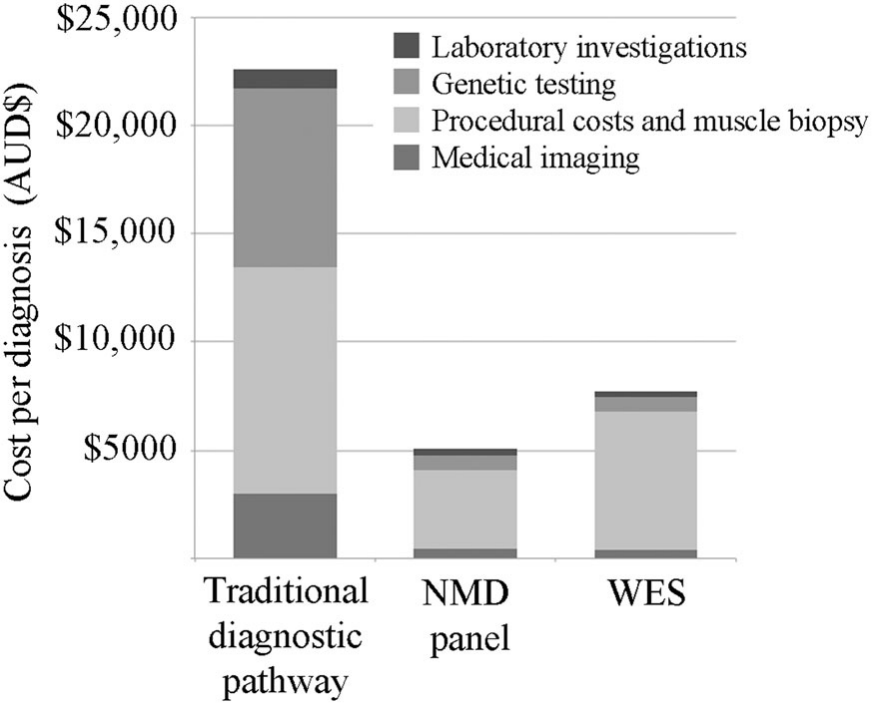
Cost savings by investigation type. Cost per diagnosis ($AUD) of the traditional diagnostic pathway compared with NMD gene panel and WES. Costs are divided by laboratory investigations, genetic testing, procedural costs, and muscle biopsy and medical imaging, demonstrating cost savings using a molecular approach to diagnosis, predominantly in genetic testing costs and procedural costs

**Fig. 4:**
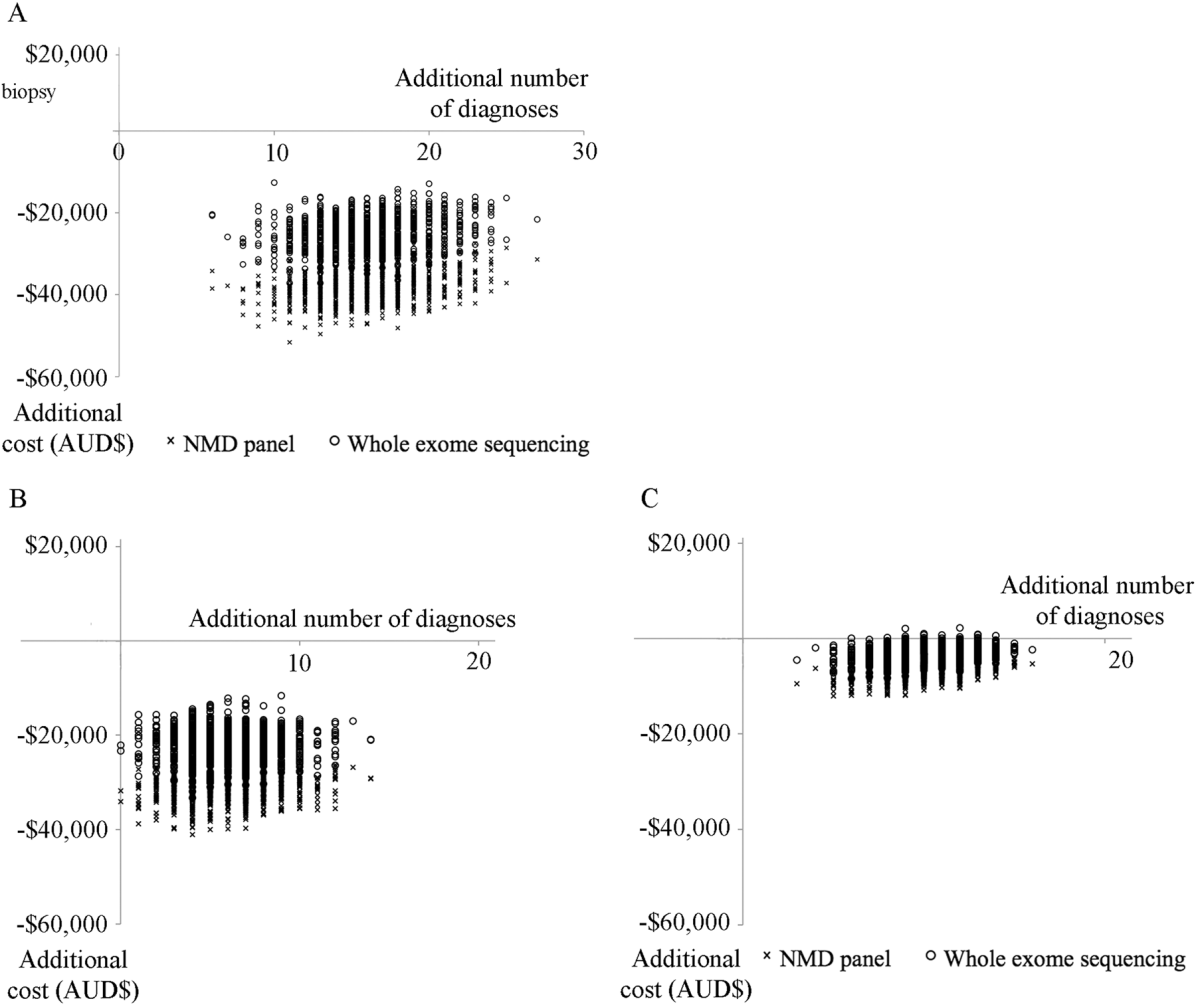
Cost-effectiveness of NMD panel or WES compared with the traditional diagnostic pathway. **a** Cost saving per additional diagnosis, for the combined CMD and NM cohort, for WES and NMD panel compared with the traditional diagnostic pathway. **b** Cost saving per additional diagnosis for the CMD cohort. **c** Cost saving per additional diagnosis for the NM cohort

A comparative analysis of the combined CMD and NM cohort was performed, representative of a patient cohort for whom there was early access to WES or panel testing, prior to muscle biopsy. While both the NMD panel and WES were superior (i.e. less costly with a higher number of diagnoses) compared to the traditional diagnostic pathway, the NMD panel was the most cost effective, with an incremental cost saving per diagnosis of AUD$23,390 (95% CI: $14,595–$41,184) compared to traditional diagnostic investigations. Although less cost-effective than the NMD panel, WES offered a cost saving per diagnosis of AUD$13,732 (95% CI: $7938–$23,473) (Supplementary Table e2). Comparative costs per diagnosis and cost-effectiveness planes are presented in Figs. 3 and 4.

The average cost of traditional diagnostic investigation was AUD$10,491 (95% CI: $9115–$11,848) per *patient*, and AUD$22,596 (95% CI: $17,004–$31,498) per successful *diagnosis*. The NMD panel provided 16 more diagnoses than traditional investigations (42/56 patients, 75%), a 28.6% (95% CI: 17.8–41.1%) increase in diagnostic rates and cost $6683 (95% CI: $5276–$7947) less per patient. The increase in diagnostic rates and the reduction in the cost per patient were statistically significant at 5% level of significance. The NMD panel cost less than a quarter of the cost of the traditional pathway per *diagnosis* AUD$5077 (95% CI: $4228–$6100). WES achieved two more diagnoses than the NMD panel at a diagnostic rate of 79% (44/56 patients), and a 32.1% (95% CI: 21.4–44.5%) increase compared to the traditional pathway and cost significantly less per patient, while costing about one-third of the cost of the traditional pathway per *diagnosis* AUD$7734 (95% CI: $6166–$9696) (Supplementary Table e2, Fig. 3).

The findings were similar when the CMD and NM cohorts were analysed separately (Supplementary Table e2). The cost-effectiveness plane for the CMD cohort is shown in Fig. 4b and for the nemaline cohort in Fig. 4c.

The costs were re-calculated for probands only. As expected, there was a slightly increased cost of diagnosis (Supplementary Table e3), reflecting the reduced cost of investigating an affected sibling with Sanger sequencing when the pathogenic variant had already been identified in the family. However, given the small number of siblings in this cohort (*n* = 6), there was not a statistically significant difference when compared with the total cohort.

## Discussion

This study demonstrates that using either a NMD panel or WES, prior to biopsy, is less costly and more effective than traditional diagnostic investigation. The diagnostic efficacy increased from 46% for traditional investigation, to 75% for the NMD panel and 79% for WES, and the total average diagnostic expenditure per *patient* was reduced from AUD$10,491 to AUD$3808 for the NMD panel and AUD$6077 for WES. The total average cost per *diagnosis* was reduced from AUD$22,596 to AUD$5077 for the NMD panel and AUD$7734 for WES.

Patients undiagnosed by traditional means were investigated with WES. The additional diagnoses in this cohort highlight some key advantages of massively parallel sequencing, namely, identification of unexpected diagnoses or a typical phenotypes, for example, the patient diagnosed with a congenital myasthenic syndrome; and variants in large genes such as *TTN*, *NEB*, and *RYR1*, which have been technically difficult and prohibitively expensive to Sanger sequence. Overall, fewer investigations were performed in the nemaline cohort as part of the traditional diagnostic work up, as the diagnosis of NM was made on muscle ultrastructural analysis and the commercial cost of *NEB* sequencing (AUD$7952.00) was not financially viable. Identification of *NEB* as the causative gene in almost half of the nemaline patients diagnosed via targeted massively parallel sequencing panel (6 of 11 patients) highlights the power of this approach for these patients.

Although WES was more expensive than NMD panel, it permits retrospective data analysis as new genes are identified and offers the potential to identify novel genetic causes of disease, particularly when coupled with a research programme. Within this cohort, two novel causes of disease (*PIGY* and *LMOD3*) were identified.^5, 6^ This study was, however, intended to reflect clinical practice, rather than a research setting, and commercial costs have therefore been selected for WES and NMD panel. We stress the importance of WES as a gene discovery research tool that underpins expansion of NMD gene panels, but note that our calculations do not include the considerable research costs involved in performing the functional studies necessary to verify pathogenicity of novel disease genes or novel genetic mechanisms (for example, splicing aberrations). Important for centres without a parallel research programme, the NMD gene panel offered an excellent diagnostic yield (16 further diagnoses) and improved cost-effectiveness when compared with the traditional diagnostic pathway (an incremental cost saving of AUD$23,390 per additional diagnosis).

Our molecular approach to diagnosis prescribes a list of limited investigations recommended prior to investigation with WES or NMD panel (Table 2), focusing on the exclusion of diagnoses not identified by these technologies. These include chromosomal microarray to exclude large-scale copy number variation, or tailored genetic testing for trinucleotide repeat disorders, not easily detected using massively parallel sequencing. In addition to cost savings, a key benefit of the molecular approach to diagnosis is avoidance of procedures in some patients. Muscle biopsy, and the associated general anaesthetic, is a potential risk in infants and children with severe weakness and impaired respiratory function, and has a risk of malignant hyperthermia reaction in some patients. However, muscle biopsy will still be required as a second-tier investigation in patients with variants in novel or recently described disease genes, and has significant utility for diagnosis of nemaline patients with variants of uncertain significance in *NEB*. Morphological confirmation of nemaline bodies on biopsy and our research-based utilisation of RNA-seq from muscle biopsy specimens have been essential in establishing pathogenicity of splice variants, particularly in *NEB* (Cummings et al., personal communication)*.*

**Table 2:** Essential and recommended investigations used for assignment of counterfactual costs

Neonatal/early infantile presentation	Early childhood presentation
*Essential investigations*	*Essential investigations*
Blood collection × 2	Blood collection × 2
Creatine kinase	Creatine kinase
Lactate	Lactate
Ammonia	Ammonia
Urine metabolic screen	Urine metabolic screen
DNA extraction and storage	DNA extraction and storage
Chromosomal microarray	Chromosomal microarray
*Investigations which might still be considered depending on presentation*	*Investigations which might still be considered depending on presentation*
Very long chain fatty acids	Thyroid function studies
Thyroid function studies	Myotonic dystrophy (DM1)
Plasma amino acids	*SMN1* exon 7 copy number (SMA)
Myotonic dystrophy	MRI brain +/− anaesthetic and day stay admission
*SMN1* exon 7 copy number (SMA)	Muscle MRI scan +/− anaesthetic and day stay admission
Prader Willi syndrome	Dystrophin MLPA
Acetylcholine receptor antibodies	Ophthalmology review
Antibody screen for congenital infection	Connective tissue dysplasia clinic review
Lumbar puncture with lactate, amino acids, and neurotransmitters	
Head ultrasound scan	
MRI brain +/− anaesthetic and day stay admission	
Ophthalmology review	

*MLPA* multiplex ligation-dependent probe amplification, *SMA* spinal muscular atrophy

In summary, our diagnostic evaluation and economic analysis provide support for molecular diagnosis using massively parallel sequencing technologies as a first-tier diagnostic investigation for paediatric NMD, replacing traditional investigation with muscle biopsy and candidate gene sequencing. WES is a valuable tool in the investigation of undiagnosed cases, particularly when coupled with a research-based gene discovery programme. A molecular approach to diagnosis also has the potential to decrease the duration of the diagnostic odyssey. Of note, five families in this study had multiple affected siblings. Early genetic diagnosis has significant implications for families and society, including informed reproductive choices, a potential impact on genetic counselling and fertility services, and the possibility that some families may chose not to have a second affected child. Quantification of these downstream impacts was outside the scope of this study, but warrants assessment in a future prospective study. This study offers evidence to support the cost-effectiveness of massively parallel sequencing techniques in the diagnosis of highly heterogeneous paediatric muscle disorders and to advocate for equitable access to genetic diagnosis in clinical practice.

## Methods

This study was a retrospective cohort analysis conducted in a publically funded Australian paediatric hospital. Patients referred with suspected CMD or NM over a 15-year period (1998–2013) were identified through the clinical records of the Neuromuscular Clinic at the Children’s Hospital at Westmead, and the Institute for Neuroscience and Muscle Research (Sydney, Australia) Biospecimen Bank. Patients referred to the clinic for a second opinion, and patients whose diagnostic evaluation had been performed elsewhere were excluded to ensure complete ascertainment of costs (*n* = 3).

CMD patients were included in the study if their clinical presentation was consistent with CMD, CK levels were elevated (>200 IU/L) and the muscle biopsy showed dystrophic changes or showed non-specific myopathic findings, provided the clinical criteria were met.^2, 7^ NM patients were included if their clinical presentation was consistent with a congenital-onset or childhood-onset myopathy and muscle biopsy showed nemaline rods on gomori trichrome stain and/or electron microscopy.^8^

Patients had been investigated using a traditional diagnostic approach^2, 7, 8^ (Fig. 1), which routinely included measurement of CK, muscle biopsy, and histological analysis. Further investigations were performed at the discretion of the treating clinician according to the patient’s phenotype. Patients who remained undiagnosed despite candidate gene sequencing were offered WES or a massively parallel sequencing-based neuromuscular gene panel (NMD panel). WES was performed by the Broad Institute, on an Illumina HiSeq2000 and/or 2500s, using a previously published protocol.^9^ The NMD panel represents a commercially available 464 neuromuscular gene panel offered by PathWest Laboratory, Australia (Supplementary Table e4). The duration of the diagnostic trajectory was calculated as the time from onset of symptoms until a genetic diagnosis was reported, or an uninformative WES report was issued.

Approval for all aspects of this study was obtained from the Human Research Ethics Committee of the Sydney Children’s Hospitals Network (Approval No: 10.CHW.45). Written informed consent was obtained from all participants for further genetic testing.

### Economic analysis

Economic analysis was performed from the payers’ perspective and covered the period from the referral of patients with suspected CMD or NM to a successful genetic diagnosis with one of the pathways (the traditional diagnostic pathway, the NMD panel, or WES) or the return of the WES results if no genetic diagnosis was achieved. Cost data reflect the 2016 commercial charges to the hospital for all diagnostic investigations and procedures, extracted from the medical records of each patient. The current cost was identified either from the Australian Medicare Benefits Schedule, or from the hospital, research, or commercial laboratory which provided the test (Supplementary Table e5). These costs include sequencing plus bioinformatics and analysis. The cost of regular clinic assessment was not included, as patients were seen regularly in clinic regardless of whether or not they had a genetic diagnosis. Geneticists and genetic counsellors work routinely in our clinic and were available to all patients. Genetic counselling for families undergoing WES potentially requires additional resources, but could not be quantified in this analysis. Costs in overseas currencies were converted into Australian dollars (AUD$) (AUD$1 = USD$0.73 = GBP£0.50; Jan 2016).

We compared the traditional diagnostic pathway with two alternative diagnostic pathways (counterfactual pathways), either NMD gene panel or WES. The counterfactual pathways assumed molecular investigation with NMD gene panel or WES prior to muscle biopsy (Fig. 1). Table 2 details a list of investigations recommended prior to the NMD panel or WES diagnostic approach. Supplementary Tables e2 and e4 detail the costs of investigations.

1. Traditional diagnostic pathway: Costing includes all diagnostic investigations, procedures, and assessments for the traditional diagnostic assessment, followed by Sanger sequencing of candidate genes in genomic DNA or mRNA/cDNA extracted from biopsy specimens, and confirmation of segregation of dominant and recessive variant(s) in gDNA extracted from the parents.
2. NMD gene panel: Costing includes recommended investigations (Table 2), if these investigations had been performed as part of the traditional diagnostic pathway for the proband, DNA extraction, and national shipping, a commercially available 464 gene neuromuscular panel (PathWest Laboratory, Australia, AUD$1100.00) (Supplementary Table e4), Sanger sequencing of identified causal variant(s) in the proband, and confirmation of segregation of dominant and recessive variant(s) in the parents.
3. WES: Costing includes recommended investigations (Table 2) if performed on the proband as per the traditional diagnostic pathway, DNA extraction and international shipment, Sanger confirmation of the identified causal variant(s) in the proband, and confirmation of segregation of dominant and recessive variant(s) in the parents. The cost of singleton WES was included if the resulting genetic diagnosis was a likely pathogenic variant in a known NMD gene, which would be detected by the NMD panel. The cost of “trio” WES was included for patients who remained undiagnosed and for patients with diagnoses made by WES if the pathogenic gene was not included in the NMD panel (i.e., Family 4 with variants in *PIGY*). The cost for WES was based on an average commercially available price of AUD$2600.00 for a singleton or AUD$7100 for a trio. Of note, the cost of a research programme necessary to take the novel genetic findings to publication is not included in this calculation.

An increased cost of Sanger sequencing was used for patients with NM due to variants in nebulin (*NEB)*, reflecting the likely need to check segregation of a minimum of three candidate variants in these patients, as *NEB* is highly polymorphic and individuals commonly have multiple Class 3 variants. No value for WES or NMD panel was included for siblings who could be diagnosed by Sanger sequencing of the known familial variant(s), or for the two patients with microdeletions detectable by chromosomal microarray.

We calculated the average cost per patient, average cost per genetic diagnosis, and incremental cost per additional diagnosis that was achieved by the NMD panel and WES diagnostic pathways compared with the traditional diagnostic pathway. To estimate the uncertainty associated with outcomes, we created 1000 replicated data sets using bootstrap simulations and estimated 95% confidence intervals (CIs) for the outcomes using the percentile method.^10^ Results are presented as scatterplots on a cost-effectiveness plane. All analyses were performed in Microsoft Excel except for bootstrap simulations and 95% CIs which were calculated in SAS version 9.4.

In calculating the diagnostic rates for WES and NMD panel diagnostic pathways, for economic evaluation, two assumptions needed to be made. It was not financially viable to re-investigate all cases previously diagnosed by candidate gene sequencing using WES or the NMD panel. It was thus assumed that all diagnoses in known CMD or myopathy genes previously identified using candidate gene sequencing would be detected by WES or the NMD panel. It was not viable to investigate undiagnosed patients with both WES and NMD panel, and it was therefore assumed that all patients diagnosed by WES would have been diagnosed using the NMD panel if the gene is included in the panel; and patients diagnosed by NMD panel would be diagnosed by WES. These assumptions are based on our previous studies which have demonstrated comparable efficacy of WES and the NMD panel for identifying variants in known NMD genes.^2, 9^

## Electronic supplementary material

Supplementary Information
