# Supplementary material for: Cost-effectiveness of massively parallel sequencing for diagnosis of paediatric muscle diseases
Source: NPJ Genom Med. 2017 Mar 3;2:4. doi: 10.1038/s41525-017-0006-7 (PMC5677979; doi:10.1038/s41525-017-0006-7)
Supplement: Supplementary file 1 — Supplementary Information [file 41525_2017_6_MOESM1_ESM.docx]

| **Table e1: Patient demographic and genetic characteristics** | | | | | | | |
| --- | --- | --- | --- | --- | --- | --- | --- |
| **Family,**  **sex** | **Consan-guinity** | **Age at onset*** | **Age at diagnosis*** | **Cohort** | **Diagnosis** | **Inheritance** | **Mode of Diagnosis** |
| 21, M | Y | 6 mo | 2y | CMD | *COL6A1* | AR | Candidate gene sequencing |
| 25, M | N | Birth | 4y | CMD | *COL6A1*  *COL6A2* | De novo AD  Het. AR variant | Candidate gene sequencing |
| 13, F | N | Birth | 10y | CMD | *COL6A2* | De novo AD | Candidate gene sequencing |
| 20, M | N | 6 mo | 13y | CMD | *COL6A2* | De novo AD | Candidate gene sequencing |
| 18, M | N | Birth | 2y | CMD | *COL6A3* | De novo AD | Candidate gene sequencing |
| 11, M | Y | 2 y | 6y | CMD | *COL6A3* | De novo AD | Candidate gene sequencing |
| 22, M | N | Birth | 4y | CMD | *LAMA2* | AR | Candidate gene sequencing |
| 36, F, F, M | N | Birth | 18mo | CMD | *LAMA2* | AR | Candidate gene sequencing |
| 6, F | N | 16 mo | 11y | CMD | *LMNA* | De novo AD | Candidate gene sequencing |
| 1, F | N | 6 mo | 11y | CMD | *POMGNT1* | AR | Candidate gene sequencing |
| 8, M, F | Y | 6 mo | 13y | CMD | *SEPN1* | AR | Candidate gene sequencing |
| 3, M | N | 6 mo | 17y | CMD | *RYR1* | AR | Candidate gene sequencing |
| 5, M | N | Birth | 26y | CMD | *RYR1* | AR | Candidate gene sequencing |
| 9, M | N | Birth | 15y | CMD | *DNM2* | De novo AD | Candidate gene sequencing |
| 12, F | N | Birth | 5y | CMD | *DNM2* | De novo AD | Candidate gene sequencing |
| 43, F | N | Birth | 3y | NM | *ACTA1*^a^ | De novo AD | Candidate gene sequencing |
| 44, M | N | Birth | 4mo | NM | *ACTA1* | De novo AD | Candidate gene sequencing |
| 47, M | N | Birth | 2mo | NM | *ACTA1* | AD^b^ | Candidate gene sequencing |
| 48, M | N | Birth | 6y | NM | *NEB* | AR | Candidate gene sequencing |
| 49, F | N | Birth | 3y^+^ | NM | *NEB* | AR | Candidate gene sequencing + dHPLC |
| 50, M ^c^ | N | 3y | 8yr | NM | *TPM2* | AD | Candidate gene sequencing |
| 15, M | N | Birth | 13y | CMD | Microdeletion 14q13.2-q21.1 | De novo AD | Microarray |
| 17, M | N | Birth | 10y | CMD | Microdeletion 6q13-q14.1 | De novo AD | Microarray |
| 33, M | N | Birth | 3y | CMD | *ACTA1* | De novo AD | WES proband only |
| 42, F | N | 2mo | 10y^+^ | NM | *ACTA1* | De novo AD (mosaic?) | WES proband and parents |
| 10, F | N | Birth | 11y | CMD | *GFPT1* | AR | WES proband and parents |
| 37, F, F | N | Birth | 9y | NM | *LMOD3* | AR | WES proband, sibling, parents |
| 27, M | N | 1y | 7y | CMD | *MICU1* | AR | WES proband and parents |
| 38, M | N | Birth | 18y | NM | *NEB* | AR | WES proband and parents; NMD panel proband |
| 45, F, M | Y | 1y | 23y | NM | *NEB* | AR | WES proband; NMD panel proband |
| 46, F | N | Birth | 4y | NM | *NEB* | AR | WES proband and parent |
| 4, F, F | N | Birth | 14y^+^ | CMD | *PIGY* | AR | WES proband, sibling and parents |
| 41, F | N | Birth | 8y | NM | *PLOD1* | AR | WES proband and parents |
| 26, F | N | 18 mo | 6y | CMD | *POMT1* | AR | WES proband and parents |
| 28, M | N | 3 mo | 3y | CMD | *RYR1* | AR | WES proband and parents |
| 23, M | N | 6 mo | 10y | CMD | *TTN*^d^ | AR | WES proband and parents |
| 51, F | N | Birth | 5mo | NM | *ACTA1*^e^ | De novo AD | NMD panel |
| 52, F | N | Birth | 4mo | NM | *ACTA1* | De novo AD | NMD panel |
| 7, M | N | Birth |  | CMD |  |  |  |
| 2, M | Y | Birth |  | CMD |  |  |  |
| 19, M | N | Birth |  | CMD |  |  |  |
| 24, M | N | 12 mo |  | CMD |  |  |  |
| 29, F | N | Birth |  | CMD |  |  |  |
| 30, M | N | 1 y |  | CMD |  |  |  |
| 31, M | N | Birth |  | CMD |  |  |  |
| 32, F | N | 6 mo |  | CMD |  |  |  |
| 34, F | N | 1 y |  | CMD |  |  |  |
| 35, F | N | 2.5 y |  | CMD |  |  |  |
| 39, M | N | Birth |  | NM |  |  |  |
| 40, F | N | 2mo | 14y | NM | *NEB* | AR | RNAseq proband^f^ |
| 14, M | Y | 7 mo |  | CMD |  |  | Declined genetic testing^g^ |
| 16, M | N | Birth |  | CMD |  |  | No consent for NGS^g^ |
| * Proband; ^+^ Patient deceased – chronological age if patient were alive at the time of diagnosis; ^a^ Phenotype and histology consistent with NM. Compound heterozygous variants in *KLHL40* may also contribute to phenotype; ^b^ Mother mosaic for the variant identified, but clinically unaffected; ^c^ Mother also affected; ^d^ Phenotype and histology consistent with *TTN*-related myopathy. Pathogenicity of Class III variants unable to be confirmed. ^e^ Clinical features consistent with *ACTA1*-related congenital myopathy. Deceased prior to muscle biopsy; ^f^ Compound heterozygous variants in *NEB* identified on RNAseq. These were not identified on NMD panel or WES and the patient was classified as undiagnosed for the purposes of this study; ^g^ Excluded from further analysis.  AD, autosomal dominant; AR, autosomal recessive; CMD, congenital muscular dystrophy; NGS, next generation sequencing; NM, nemaline myopathy; WES, whole exome sequencing; dHPLC, Denaturing high pressure liquid chromatography. | | | | | | | |

**Supplementary Information**

| **Table e2. Cost of standard diagnostic care compared with investigation with NMD gene panel or WES** | | | | | | | | | |
| --- | --- | --- | --- | --- | --- | --- | --- | --- | --- |
|  | **Congenital MD Cohort** (n=38) | | | **Nemaline myopathy cohort** (n=18) | | | **Combined cohorts** (n=56) | | |
| **Investigation** | **Standard** | **NMD panel** | **WES** | **Standard** | **NMD panel** | **WES** | **Standard** | **NMD panel** | **WES** |
| **Laboratory investigations** | | | | | | | | | |
| Blood collection | 6540 | 2250 | 2250 | 1740 | 960 | 960 | 8280 | 3210 | 3210 |
| Creatine kinase | 1684.3 | 701.75 | 701.75 | 240.6 | 240.6 | 240.6 | 1924.9 | 942.35 | 942.35 |
| Ammonia | 135.8 | 116.4 | 116.4 | 38.8 | 38.8 | 38.8 | 174.6 | 155.2 | 155.2 |
| Lactate | 335.1 | 203.7 | 203.7 | 48.5 | 48.5 | 48.5 | 383.6 | 252.2 | 252.2 |
| Thyroid function tests | 826.65 | 475.95 | 475.95 | 75.15 | 75.15 | 75.15 | 901.8 | 551.1 | 551.1 |
| TORCH screen | 222.8 | 167.1 | 167.1 | 0 | 0 | 0 | 222.8 | 167.1 | 167.1 |
| Metabolic studies^1^ and metabolic review | 20184.85 | 917.6 | 917.6 | 5698.08 | 3981.7 | 3981.7 | 25882.93 | 4899.3 | 4899.3 |
| Urine metabolic screen | 5000 | 3800 | 3800 | 2000 | 2000 | 2000 | 7000 | 5800 | 5800 |
| Mitochondrial studies^2^ | 14110.08 | 0 | 0 | 0 | 0 | 0 | 14110.08 | 0 | 0 |
| ANA, anti-ds DNA, ENA screen | 68.35 | 0 | 0 | 0 | 0 | 0 | 68.35 | 0 | 0 |
| AChR and MUSK Abs | 387.2 | 69.1 | 69.1 | 343.65 | 343.65 | 343.65 | 721.85 | 412.75 | 412.75 |
| Fibroblast culture | 10500 | 0 | 0 | 5250 | 1050 | 1050 | 15750 | 1050 | 1050 |
| Fibroblast retrieval | 1920 | 0 | 0 | 0 | 0 | 0 | 1920 | 0 | 0 |
| Myoblast culture | 0 | 0 | 0 | 800 | 400 | 400 | 800 | 400 | 400 |
|  | **61915.13** | **8701.6** | **8701.6** | **16234.78** | **9138.4** | **9138.4** | **78140.91** | **17840** | **17840** |
| **Genetic testing** | | | | | | | | | |
| Chromosomal microarray | 13238 | 15007.7 | 15007.7 | 3539.4 | 3539.4 | 3539.4 | 16777.4 | 18547.1 | 18547.1 |
| Candidate gene sequencing | 125453.76 | 0 | 0 | 54384 | 0 | 0 | 179837.76 | 0 | 0 |
| Other genetic testing^3^ | 15163.95 | 4340 | 4340 | 12964.5 | 12130.55 | 12130.55 | 28128.45 | 16470.55 | 16470.55 |
| DNA extraction and sendaway costs | 13280 | 9180 | 10170 | 7290 | 5530 | 5980 | 20570 | 14710 | 16150 |
| Blood collection, DNA extraction and sendaway (parents and siblings) | 7230 | 10110 | 14520 | 3050 | 6100 | 6220 | 10280 | 16210 | 20740 |
| NMD gene panel | 0 | 36300 | 0 | 0 | 17600 | 0 | 0 | 53900 | 0 |
| WES - proband | 0 | 0 | 59800 | 0 | 0 | 36400 | 0 | 0 | 96200 |
| WES - trio | 0 | 0 | 71000 | 0 | 0 | 14200 | 0 | 0 | 85200 |
| Confirmation sequencing | 13400 | 20200 | 13800 | 3200 | 14400 | 14400 | 16600 | 34600 | 28200 |
|  | **187765.71** | **95137.7** | **188637.7** | **84427.9** | **59299.95** | **92869.95** | **272193.61** | **154437.65** | **281507.65** |
| **Procedural costs and anatomical pathology** | | | | | | | | | |
| Muscle biopsy, histology and EM | 12544.7 | 0 | 0 | 6963.95 | 3753^4^ | 3753^4^ | 20508.65 | 3753 | 3753 |
| IHC and western blot analysis | 41000 | 0 | 0 | 550 | 0 | 0 | 41550 | 0 | 0 |
| Nerve biopsy and histology | 442.55 | 0 | 0 | 885.1 | 0 | 0 | 1327.65 | 0 | 0 |
| Skin biopsy | 1618.2 | 0 | 0 | 679.2 | 52.5 | 52.5 | 2297.4 | 52.5 | 52.5 |
| Suction rectal biopsy | 224.60 | 0 | 0 | 0 | 0 | 0 | 224.60 | 0 | 0 |
| EM fibroblasts | 245.8 | 0 | 0 | 0 | 0 | 0 | 245.8 | 0 | 0 |
| EEG | 1046.2 | 0 | 0 | 0 | 0 | 0 | 1046.2 | 0 | 0 |
| Electrophysiology | 11083.95 | 0 | 0 | 3359.5 | 0 | 0 | 14443.45 | 0 | 0 |
| Admission and sedation/ anaesthetic costs for procedures | 90782.18 | 5248.14 | 5248.14 | 34038.69 | 12608.66 | 12608.66 | 124820.87 | 17856.8 | 17856.8 |
| Connective tissue dysplasia clinic review | 342.2 | 171.1 | 171.1 | 0 | 0 | 0 | 342.2 | 171.1 | 171.1 |
| Ophthalmology review | 771.2 | 771.2 | 771.2 | 192.8 | 192.8 | 192.8 | 964 | 964 | 964 |
| Post mortem | 0 | 0 | 0 | 6200 | 6200 | 6200 | 6200 | 6200 | 6200 |
|  | **160101.58** | **6190.44** | **6190.44** | **52869.24** | **22806.96** | **22806.96** | **213970.82** | **28997.4** | **28997.4** |
| **Medical Imaging** | | | | | | | | | |
| MRI brain or spine | 9273.4 | 5241.6 | 5241.6 | 6227.2 | 4972.8 | 4972.8 | 15500.6 | 10214.4 | 10214.4 |
| Muscle imaging | 2992.9 | 0 | 0 | 599 | 599 | 599 | 3591.9 | 599 | 599 |
| Anaesthetic for MRI | 2910.6 | 415.8 | 415.8 | 415.8 | 415.8 | 415.8 | 3326.4 | 831.6 | 831.6 |
| Head ultrasound scan | 218.2 | 163.65 | 163.65 | 163.65 | 163.65 | 163.65 | 381.85 | 327.3 | 327.3 |
| Skeletal survey | 111.3 | 0 | 0 | 0 | 0 | 0 | 111.3 | 0 | 0 |
| Abdominal ultrasound | 268.2 | 0 | 0 | 0 | 0 | 0 | 268.2 | 0 | 0 |
|  | **15774.6** | **5821.05** | **5821.05** | **7405.65** | **6151.25** | **6151.25** | **23180.25** | **11972.3** | **11972.3** |
| **Total cost ($AUD)**  (CMD=38; NM=18) | **426548.02** | **115850.79** | **209350.79** | **160937.57** | **97396.56** | **130966.56** | **587485.59** | **213247.35** | **340317.35** |
| **Total number of diagnoses made** | 20 | 26 | 28 | 6 | 16 | 16 | 26 | 42 | 44 |
| **Mean cost per patient**  **(95%CI)** | 11224.95  (9584.38, 12927.92) | 3048.71  (2640.83, 3463.13) | 5509.23  (4621.92, 6301.18) | 8940.98  (6627.44, 11355.81) | 5410.92  (4322.95, 6463.57) | 7275.92  (5844.37, 8645.70) | 10490.81  (9114.91, 11847.74) | 3807.99  (3293.13, 4373.41) | 6077.10  (5284.05, 6846.44) |
| **Standard deviation** | 5395.91 | 1331.66 | 2778.59 | 5220.99 | 2525.68 | 3112.77 | 5401.01 | 2098.54 | 2980.25 |
| **Mean cost per diagnosis**  (95%CI) | 21327.40  (15700.57, 30774.11) | 4455.80  (3596.81, 5626.48) | 7476.81  (5448.62, 10355.36) | 26822.93  (15908.56, 73195.83) | 6087.29  (4693.59, 7883.40) | 8185.41  (6128.19, 11355.59) | 22595.60  (17003.56, 31498.03) | 5077.32  (4228.10, 6099.99) | 7734.49  (6165.98, 9696.07) |
| **Incremental saving per additional diagnosis**  **(95%CI)** | - | 51782.87  (27185.58, 166520.27) | 27149.65  (14682.77, 66614.64) | - | 6354.10  (2490.28, 14906.07) | 2997.10  (111.45, 8450.70) | - | 23389.89  (14594.86,  41183.99) | 13731.57  (7937.58,23472.53) |
| 1. 7 dehydrocholesterol, 8-dehydrocholesterol and 8(9) cholestanol studies on fibroblasts, bile alcohol analysis, carnitine palmitoyl transferase II, acid maltase deficiency, acylcarnitine profile, CDG1A and 1B analysis on white blood cells, copper, ceruloplasmin, cholesterol and triglycerides, conenzyme Q10 analysis, creatine and guanadinoacetate studies, DHAP-AT and DHAP-s studies, fibroblast acylcarnitine studies, homocysteine, methylmalonate, neuraminidase levels, transferrin isoforms, white cell enzymes, very long chain fatty acids, uric acid, pyruvate, phytanic acid, plasma amino acids, plasmalogens, urine oligosaccharides, urine purines and pyrimidines, lumbar puncture and CSF studies: microscopy, culture, protein, glucose, lactate, amino acids, neurotransmitters 2. Liver biopsy and sendaway, mitochondrial enzyme analysis, depletion studies, mitochondrial genome sequencing 3. SMN1 deletion, myotonic dystrophy, Fragile X, Prader Willi, subtelomeric FISH studies incl 22q11.2, dystrophin MLPA and sequencing, standard karyotype, FISH studies, facioscapular humeral dystrophy 4. Muscle biopsy was required in some patients to look for nemaline rods on biopsy and to assess the pathogenicity of splice site variants in *NEB* using RNA sequencing   ANA, antinuclear antibodies; AChR Abs, Acetylcholine receptor antibodies; MUSK Abs, Muscle specific kinase antibodies; EM, electron microscopy; IHC, immunohistochemistry; EEG, electroencephalogram; NMD, neuromuscular disease; WES, whole exome sequencing | | | | | | | | | |

| **Table e3. Cost of standard diagnostic care compared with investigation with NMD gene panel or WES**  **Cohort with affected siblings excluded** | | | | | | | | | | |
| --- | --- | --- | --- | --- | --- | --- | --- | --- | --- | --- |
|  | **Congenital MD Cohort** (n=34) | | | **Nemaline myopathy cohort** (n=16) | | | | **Combined cohorts** (n=50) | | |
| **Investigation** | **Standard** | **NMD panel** | **WES** | **Standard** | **NMD panel** | **WES** | **Standard** | | **NMD panel** | **WES** |
| **Total Cost ($AUD)**  (CMD=34; NM=16) | 411632.1 | 112651.7 | 204621.7 | 159321.6 | 96276.51 | 129846.5 | 570953.7 | | 208928.3 | 334468.3 |
| **Total number of diagnoses made** | 17 | 23 | 24 | 6 | 14 | 14 | 23 | | 37 | 38 |
| **Mean cost per patient**  (95%CI) | 12106.83  (10569.81, 13739.34) | 3313.29  (2945.83, 3688.51) | 6018.29  (5217.03, 6804.55) | 9957.60  (7865.02, 12247.79) | 6017.28  (5081.50, 6928.30) | 8115.41  (7058.38, 9102.06) | 11419.07  (10239.09, 12812.36) | | 4178.57  (3636.57, 4719.28) | 6689.37  (6035.21, 7339.78) |
| **Standard deviation** | 4636.97 | 1134.30 | 2448.04 | 4579.00 | 1923.24 | 2052.97 | 4682.39 | | 1903.23 | 2510.53 |
|  |  |  |  |  |  |  |  | |  |  |
| **Mean cost per diagnosis**  (95%CI) | 24213.66  (18371.48, 35930.13) | 4897.90  (4005.12, 6281.79) | 8525.91  (6441.00, 12094.56) | 26553.60  (15975.9, 74778.59) | 6876.89  (5581.42, 8599.77) | 9274.75  (7328.26, 13045.11) | 24824.07  (19104.67, 36026.67) | | 5646.71  (4840.42, 6595.35) | 8801.80  (7154.96, 10830.02) |
| **Incremental saving per additional diagnosis** (95%CI) | - | 49830.07  (26040.07-160081.85) | 29572.91  (15856.94-79634.05) | - | 7880.63  (3226.60,20169.91) | 3684.38  (143.16-11363.11) | - | | 25858.96  (15799.95, 48284.14) | 15765.70  (9168.80,28835.76) |
| CMD, congenital muscular dystrophy; NMD, neuromuscular disease; NM, nemaline myopathy; WES, whole exome sequencing | | | | | | | | | | |

| **Table e4: Genes included in the PathWest Neuromuscular Gene Panel** | | | | | |
| --- | --- | --- | --- | --- | --- |
| **Gene Name** | **HGNC_ID** | **Description** | **Gene Name** | **HGNC_ID** | **Description** |
| AARS | 20 | Alanyl-tRNA synthetase | KIF21A | 19329 | kinesin family member 21A |
| ABCC9 | 60 | ATP-binding cassette, sub-family C (CFTR/MRP), member 9 | KIF5A | 6323 | kinesin family member 5A |
| ABCD1 | 61 | ATP-binding cassette, sub-family D (ALD), member 1 | KLHL9 | 18732 | kelch-like family member 9 |
| ABHD12 | 15868 | abhydrolase domain containing 12 | L1CAM | 6470 | L1 cell adhesion molecule |
| ABHD5 | 21396 | abhydrolase domain containing 5 | LAMA2 | 6482 | laminin, alpha 2 |
| ACADVL | 92 | acyl-CoA dehydrogenase, very long chain | LAMA4 | 6484 | laminin, alpha 4 |
| ACTA1 | 129 | actin, alpha 1, skeletal muscle | LAMB2 | 6487 | laminin, beta 2 (laminin S) |
| ACTC1 | 143 | actin, alpha, cardiac muscle 1 | LAMP2 | 6501 | lysosomal-associated membrane protein 2 |
| ACTN2 | 164 | actinin, alpha 2 | LARGE | 6511 | like-glycosyltransferase |
| ACVR1 | 171 | activin A receptor, type I | LDB3 | 15710 | LIM domain binding 3 |
| ADCK3 | 16812 | aarF domain containing kinase 3 | LDHA | 6535 | lactate dehydrogenase A |
| AFG3L2 | 315 | AFG3-like AAA ATPase 2 | LITAF | 16841 | lipopolysaccharide-induced TNF factor |
| AGL | 321 | amylo-alpha-1, 6-glucosidase, 4-alpha-glucanotransferase | LMNA | 6636 | lamin A/C |
| AGRN | 329 | agrin | LMOD3 | 6649 | leiomodin 3 (fetal) |
| AHNAK | 347 | AHNAK nucleoprotein | LPIN1 | 13345 | lipin 1 |
| AIFM1 | 8768 | apoptosis-inducing factor, mitochondrion-associated, 1 | LRSAM1 | 25135 | leucine rich repeat and sterile alpha motif containing 1 |
| ALDH3A2 | 403 | aldehyde dehydrogenase 3 family, member A2 | MATR3 | 6912 | matrin 3 |
| ALS2 | 443 | amyotrophic lateral sclerosis 2 (juvenile) | MED25 | 28845 | mediator complex subunit 25 |
| ANG | 483 | angiogenin, ribonuclease, RNase A family, 5 | MEGF10 | 29634 | multiple EGF-like-domains 10 |
| ANK2 | 493 | ankyrin 2, neuronal | MFN2 | 16877 | mitofusin 2 |
| ANKRD1 | 15819 | ankyrin repeat domain 1 (cardiac muscle) | MPZ | 7225 | myelin protein zero |
| ANO10 | 25519 | anoctamin 10 | MRE11A | 7230 | MRE11 homolog A, double strand break repair nuclease |
| ANO5 | 27337 | anoctamin 5 | MRPL3 | 10379 | mitochondrial ribosomal protein L3 |
| AP5Z1 | 22197 | adaptor-related protein complex 5, zeta 1 subunit | MSTN | 4223 | myostatin |
|  |  |  | MTM1 | 7448 | myotubularin 1 |
| APOA1 | 600 | apolipoprotein A-I | MTMR2 | 7450 | myotubularin related protein 2 |
| APTX | 15984 | aprataxin | MTTP | 7467 | microsomal triglyceride transfer protein |
| AR | 644 | androgen receptor | MURC | 33742 | muscle-related coiled-coil protein |
| ARHGEF10 | 14103 | Rho guanine nucleotide exchange factor (GEF) 10 | MUSK | 7525 | muscle, skeletal, receptor tyrosine kinase |
| ARSA | 713 | arylsulfatase A | MYBPC1 | 7549 | myosin binding protein C, slow type |
| ARX | 18060 | aristaless related homeobox | MYBPC3 | 7551 | myosin binding protein C, cardiac |
| ASAH1 | 735 | N-acylsphingosine amidohydrolase (acid ceramidase) 1 | MYH2 | 7572 | myosin, heavy chain 2, skeletal muscle, adult |
| ATL1 | 11231 | atlastin GTPase 1 | MYH3 | 7573 | myosin, heavy chain 3, skeletal muscle, embryonic |
| ATM | 795 | ATM serine/threonine kinase | MYH6 | 7576 | myosin, heavy chain 6, cardiac muscle, alpha |
| ATP2A1 | 811 | ATPase, Ca++ transporting, cardiac muscle, fast twitch 1 | MYH7 | 7577 | myosin, heavy chain 7, cardiac muscle, beta |
| ATP2B3 | 816 | ATPase, Ca++ transporting, plasma membrane 3 | MYH8 | 7578 | myosin, heavy chain 8, skeletal muscle, perinatal |
| ATP7A | 869 | ATPase, Cu++ transporting, alpha polypeptide | MYL2 | 7583 | myosin, light chain 2, regulatory, cardiac, slow |
| B3GALNT2 | 28596 | beta-1,3-N-acetylgalactosaminyltransferase 2 | MYL3 | 7584 | myosin, light chain 3, alkali; ventricular, skeletal, slow |
| BAG3 | 939 | BCL2-associated athanogene 3 | MYLK2 | 16243 | myosin light chain kinase 2 |
| BEAN1 | 24160 | brain expressed, associated with NEDD4, 1 | MYOT | 12399 | myotilin |
| BIN1 | 1052 | bridging integrator 1 | MYOZ2 | 1330 | myozenin 2 |
| BSCL2 | 15832 | Berardinelli-Seip congenital lipodystrophy 2 (seipin) | MYPN | 23246 | myopalladin |
| C10orf2 | 1160 | chromosome 10 open reading frame 2 | NDRG1 | 7679 | N-myc downstream regulated 1 |
| CACNA1A | 1388 | calcium channel, voltage-dependent, P/Q type, alpha 1A subunit | NDUFAF1 | 18828 | NADH dehydrogenase (ubiquinone) complex I, assembly factor 1 |
| CACNA1C | 1390 | calcium channel, voltage-dependent, L type, alpha 1C subunit | NEB | 7720 | nebulin |
| CACNA1S | 1397 | calcium channel, voltage-dependent, L type, alpha 1S subunit | NEFL | 7739 | neurofilament, light polypeptide |
| CACNB2 | 1402 | calcium channel, voltage-dependent, beta 2 subunit | NEXN | 29557 | nexilin (F actin binding protein) |
| CACNB4 | 1404 | calcium channel, voltage-dependent, beta 4 subunit | NGF | 7808 | nerve growth factor (beta polypeptide) |
| CAPN3 | 1480 | calpain 3 | NIPA1 | 17043 | non imprinted in Prader-Willi/Angelman syndrome 1 |
| CASQ2 | 1513 | calsequestrin 2 (cardiac muscle) | NOTCH3 | 7883 | notch 3 |
| CAV3 | 1529 | caveolin 3 | NPPA | 7939 | natriuretic peptide A |
| CCT5 | 1618 | chaperonin containing TCP1, subunit 5 (epsilon) | NTRK1 | 8031 | neurotrophic tyrosine kinase, receptor, type 1 |
| CFL2 | 1875 | cofilin 2 (muscle) | OPA1 | 8140 | optic atrophy 1 (autosomal dominant) |
| CHAT | 1912 | choline O-acetyltransferase | PABPN1 | 8565 | poly(A) binding protein, nuclear 1 |
| CHRNA1 | 1955 | cholinergic receptor, nicotinic, alpha 1 (muscle) | PAFAH1B1 | 8574 | platelet-activating factor acetylhydrolase 1b, regulatory subunit 1 (45kDa) |
| CHRNB1 | 1961 | cholinergic receptor, nicotinic, beta 1 (muscle) | PDK3 | 8811 | pyruvate dehydrogenase kinase, isozyme 3 |
| CHRND | 1965 | cholinergic receptor, nicotinic, delta (muscle) | PEX7 | 8860 | peroxisomal biogenesis factor 7 |
| CHRNE | 1966 | cholinergic receptor, nicotinic, epsilon (muscle) | PFKM | 8877 | phosphofructokinase, muscle |
| CHRNG | 1967 | cholinergic receptor, nicotinic, gamma (muscle) | PFN1 | 8881 | profilin 1 |
| CLCN1 | 2019 | chloride channel, voltage-sensitive 1 | PGAM2 | 8889 | phosphoglycerate mutase 2 (muscle) |
| CNBP | 13164 | CCHC-type zinc finger, nucleic acid binding protein | PGK1 | 8896 | phosphoglycerate kinase 1 |
| CNTN1 | 2171 | contactin 1 | PGM1 | 8905 | phosphoglucomutase 1 |
| COL6A1 | 2211 | collagen, type VI, alpha 1 | PHKA1 | 8925 | phosphorylase kinase, alpha 1 (muscle) |
| COL6A2 | 2212 | collagen, type VI, alpha 2 | PHOX2A | 691 | paired-like homeobox 2a |
| COL6A3 | 2213 | collagen, type VI, alpha 3 | PHYH | 8940 | phytanoyl-CoA 2-hydroxylase |
| COLQ | 2226 | collagen-like tail subunit (single strand of homotrimer) of asymmetric acetylcholinesterase | PIP5K1C | 8996 | phosphatidylinositol-4-phosphate 5-kinase, type I, gamma |
| COX15 | 2263 | cytochrome c oxidase assembly homolog 15 (yeast) | PKP2 | 9024 | plakophilin 2 |
| CPT1B | 2329 | carnitine palmitoyltransferase 1B (muscle) | PLEC | 9069 | plectin |
| CPT2 | 2330 | carnitine palmitoyltransferase 2 | PLEKHG5 | 29105 | pleckstrin homology domain containing, family G (with RhoGef domain) member 5 |
| CRYAB | 2389 | crystallin, alpha B | PLN | 9080 | phospholamban |
| CSRP3 | 2372 | mediator complex subunit 23 | PLP1 | 9086 | proteolipid protein 1 |
| CTDP1 | 2498 | CTD (carboxy-terminal domain, RNA polymerase II, polypeptide A) phosphatase, subunit 1 | PMP22 | 9118 | peripheral myelin protein 22 |
| CYP7B1 | 2652 | cytochrome P450, family 7, subfamily B, polypeptide 1 | PNPLA2 | 30802 | patatin-like phospholipase domain containing 2 |
| DAG1 | 2666 | dystroglycan 1 (dystrophin-associated glycoprotein 1) | PNPLA6 | 16268 | patatin-like phospholipase domain containing 6 |
| DCTN1 | 2711 | dynactin 1 | POLG | 9179 | polymerase (DNA directed), gamma |
| DCX | 2714 | doublecortin | POLG2 | 9180 | polymerase (DNA directed), gamma 2, accessory subunit |
| DES | 2770 | desmin | POMGNT1 | 19139 | protein O-linked mannose N-acetylglucosaminyltransferase 1 (beta 1,2-) |
| DHTKD1 | 23537 | dehydrogenase E1 and transketolase domain containing 1 | POMT1 | 9202 | protein-O-mannosyltransferase 1 |
| DMD | 2928 | dystrophin | POMT2 | 19743 | protein-O-mannosyltransferase 2 |
| DMPK | 2933 | dystrophia myotonica-protein kinase | PRKAG2 | 9386 | protein kinase, AMP-activated, gamma 2 non-catalytic subunit |
| DNAJB2 | 5228 | DnaJ (Hsp40) homolog, subfamily B, member 2 | PRKCG | 9382 | protein kinase, cAMP-dependent, catalytic, gamma |
| DNAJB6 | 14888 | DnaJ (Hsp40) homolog, subfamily B, member 6 | PRPS1 | 9462 | phosphoribosyl pyrophosphate synthetase 1 |
| DNM2 | 2974 | dynamin 2 | PRRT2 | 30500 | proline-rich transmembrane protein 2 |
| DNMT1 | 2976 | DNA (cytosine-5-)-methyltransferase 1 | PRX | 13797 | periaxin |
| DOCK3 | 2989 | dedicator of cytokinesis 3 | PSEN1 | 9508 | presenilin 1 |
| DOK7 | 26594 | docking protein 7 | PSEN2 | 9509 | presenilin 2 |
| DPAGT1 | 2995 | dolichyl-phosphate (UDP-N-acetylglucosamine) N-acetylglucosaminephosphotransferase 1 (GlcNAc-1-P transferase) | PTRF | 9688 | polymerase I and transcript release factor |
| DPM2 | 3006 | dolichyl-phosphate mannosyltransferase polypeptide 2, regulatory subunit | PYGM | 9726 | phosphorylase, glycogen, muscle |
| DSC2 | 3036 | desmocollin 2 | RAB7A | 9788 | RAB7A, member RAS oncogene family |
| DSG2 | 3049 | desmoglein 2 | RAPSN | 9863 | receptor-associated protein of the synapse |
| DSP | 3052 | desmoplakin | RBM20 | 27424 | RNA binding motif protein 20 |
| DTNA | 3057 | dystrobrevin, alpha | REEP1 | 25786 | receptor accessory protein 1 |
| DYNC1H1 | 2961 | dynein, cytoplasmic 1, heavy chain 1 | RELN | 9957 | reelin |
| DYSF | 3097 | dysferlin | RRM2B | 17296 | ribonucleotide reductase M2 B (TP53 inducible) |
| EGR2 | 3239 | early growth response 2 | RYR1 | 10483 | ryanodine receptor 1 (skeletal) |
| EMD | 3331 | [emerin](http://www.genenames.org/cgi-bin/gene_symbol_report?hgnc_id=HGNC:3331) | RYR2 | 10484 | ryanodine receptor 2 (cardiac) |
| ENO3 | 3354 | enolase 3 (beta, muscle) | SACS | 10519 | sacsin molecular chaperone |
| ERBB3 | 3431 | erb-b2 receptor tyrosine kinase 3 | SBF2 | 2135 | SET binding factor 2 |
| ETFA | 3481 | electron-transfer-flavoprotein, alpha polypeptide | SCN4A | 10591 | sodium channel, voltage gated, type IV alpha subunit |
| ETFB | 3482 | electron-transfer-flavoprotein, beta polypeptide | SCN5A | 10593 | sodium channel, voltage gated, type V alpha subunit |
| ETFDH | 3483 | electron-transferring-flavoprotein dehydrogenase | SDHA | 10680 | succinate dehydrogenase complex, subunit A, flavoprotein (Fp) |
| EYA4 | 3522 | EYA transcriptional coactivator and phosphatase 4 | SEPN1 | 15999 | selenoprotein N, 1 |
| FA2H | 21197 | fatty acid 2-hydroxylase | SEPT9 | 7323 | septin 9 |
| FAM134B | 25964 | family with sequence similarity 134, member B | SETX | 445 | senataxin |
| FBLN5 | 3602 | Fibulin 5 | SGCA | 10805 | arcoglycan, alpha (50kDa dystrophin-associated glycoprotein) |
| FGD4 | 19125 | FYVE, RhoGEF and PH domain containing 4 | SGCB | 10806 | sarcoglycan, beta (43kDa dystrophin-associated glycoprotein) |
| FGF14 | 3671 | fibroblast growth factor 14 | SGCD | 10807 | sarcoglycan, delta (35kDa dystrophin-associated glycoprotein) |
| FGFR2 | 3689 | fibroblast growth factor receptor 2 | SGCE | 10808 | sarcoglycan, epsilon |
| FHL1 | 3702 | four and a half LIM domains 1 | SGCG | 10809 | sarcoglycan, gamma (35kDa dystrophin-associated glycoprotein) |
| FIG4 | 16873 | FIG4 phosphoinositide 5-phosphatase | SH3TC2 | 29427 | SH3 domain and tetratricopeptide repeats 2 |
| FKRP | 17997 | fukutin related protein | SIL1 | 24624 | SIL1 nucleotide exchange factor |
| FKTN | 3622 | fukutin | SLC12A6 | 10914 | solute carrier family 12 (potassium/chloride transporter), member 6 |
| FLNA | 3754 | filamin A, alpha | SLC1A3 | 10941 | solute carrier family 1 (glial high affinity glutamate transporter), member 3 |
| FLNC | 3756 | filamin C, gamma | SLC22A5 | 10969 | solute carrier family 22 (organic cation/carnitine transporter), member 5 |
| FUS | 4010 | FUS RNA binding protein | SLC25A20 | 1421 | solute carrier family 25 (carnitine/acylcarnitine translocase), member 20 |
| FXN | 3951 | frataxin | SLC25A4 | 10990 | solute carrier family 25 (mitochondrial carrier; adenine nucleotide translocator), member 4 |
| GAA | 842 | glucosidase, alpha; acid | SLC33A1 | 95 | solute carrier family 33 (acetyl-CoA transporter), member 1 |
| GAN | 4137 | gigaxonin | SMCHD1 | 29090 | structural maintenance of chromosomes flexible hinge domain containing 1 |
| GARS | 4162 | glycyl-tRNA synthetase | SMN1 | 11117 | survival of motor neuron 1, telomeric |
| GATAD1 | 29941 | GATA zinc finger domain containing 1 | SOD1 | 11179 | superoxide dismutase 1, soluble |
| GBE1 | 4180 | glucan (1,4-alpha-), branching enzyme 1 | SOX10 | 11190 | SRY-box 10 |
| GDAP1 | 15968 | ganglioside induced differentiation associated protein 1 | SPAST | 11233 | spastin |
| GFPT1 | 4241 | glutamine--fructose-6-phosphate transaminase 1 | SPG11 | 11226 | pastic paraplegia 11 (autosomal recessive) |
| GJA5 | 4279 | gap junction protein, alpha 5, 40kDa | SPG20 | 18514 | spastic paraplegia 20 (Troyer syndrome) |
| GJB1 | 4283 | gap junction protein, beta 1, 32kDa | SPG21 | 20373 | spastic paraplegia 21 (autosomal recessive, Mast syndrome) |
| GJB3 | 4285 | gap junction protein, beta 3, 31kDa | SPG7 | 11237 | spastic paraplegia 7 (pure and complicated autosomal recessive) |
| GLE1 | 4315 | GLE1 RNA export mediator | SPTBN2 | 11276 | spectrin, beta, non-erythrocytic 2 |
| GMPPB | 22932 | GDP-mannose pyrophosphorylase B | SPTLC1 | 11277 | serine palmitoyltransferase, long chain base subunit 1 |
| GNE | 23657 | glucosamine (UDP-N-acetyl)-2-epimerase/N-acetylmannosamine kinase | SPTLC2 | 11278 | serine palmitoyltransferase, long chain base subunit 2 |
| GPD1L | 28956 | glycerol-3-phosphate dehydrogenase 1-like | STIM1 | 11386 | stromal interaction molecule 1 |
| (GTDC2)  POMGNT2 | 25902 | protein O-linked mannose N-acetylglucosaminyltransferase 2 (beta 1,4-) | SUCLA2 | 11448 | succinate-CoA ligase, ADP-forming, beta subunit |
| GYG1 | 4699 | glycogenin 1 | SYNE1 | 17089 | spectrin repeat containing, nuclear envelope 1 |
| GYS1 | 4706 | glycogen synthase 1 (muscle) | SYNE2 | 17084 | spectrin repeat containing, nuclear envelope 2 |
| HARS | 4816 | histidyl-tRNA synthetase | TARDBP | 11571 | TAR DNA binding protein |
| HCN4 | 16882 | hyperpolarization activated cyclic nucleotide gated potassium channel 4 | TAZ | 11577 | tafazzin |
| HINT1 | 4912 | histidine triad nucleotide binding protein 1 | TCAP | 11610 | titin-cap |
| HK1 | 4922 | hexokinase 1 | TDP1 | 18884 | tyrosyl-DNA phosphodiesterase 1 |
| HOXD10 | 5133 | homeobox D10 | TFG | 11758 | TRK-fused gene |
| HSPB1 | 5246 | heat shock 27kDa protein 1 | TGFB3 | 11769 | transforming growth factor beta 3 |
| HSPB3 | 5248 | heat shock 27kDa protein 3 | TIA1 | 11802 | TIA1 cytotoxic granule-associated RNA binding protein |
| HSPB8 | 30171 | heat shock 22kDa protein 8 | TK2 | 11831 | thymidine kinase 2, mitochondrial |
| HSPD1 | 5261 | heat shock 60kDa protein 1 (chaperonin) | TMEM43 | 28472 | transmembrane protein 43 |
| HSPG2 | 5273 | heparan sulfate proteoglycan 2 | TMPO | 11875 | thymopoietin |
| IFRD1 | 5456 | interferon-related developmental regulator 1 | TNNC1 | 11943 | troponin C type 1 (slow) |
| IGHMBP2 | 5542 | immunoglobulin mu binding protein 2 | TNNI2 | 11946 | troponin I type 2 (skeletal, fast) |
| IKBKAP | 5959 | inhibitor of kappa light polypeptide gene enhancer in B-cells, kinase complex-associated protein | TNNI3 | 11947 | troponin I type 3 (cardiac) |
| ILK | 6040 | integrin-linked kinase | TNNT1 | 11948 | troponin T type 1 (skeletal, slow) |
| INF2 | 23791 | inverted formin, FH2 and WH2 domain containing | TNNT2 | 11949 | troponin T type 2 (cardiac) |
| ISCU | 29882 | iron-sulfur cluster assembly enzyme | TNNT3 | 11950 | troponin T type 3 (skeletal, fast) |
| ISPD | 37276 | isoprenoid synthase domain containing | TNPO3 | 17103 | transportin 3 |
| ITGA7 | 6143 | integrin, alpha 7 | TOR1A | 3098 | torsin family 1, member A (torsin A) |
| ITPR1 | 6180 | inositol 1,4,5-trisphosphate receptor, type 1 | TPM1 | 12010 | tropomyosin 1 (alpha) |
| JPH2 | 14202 | junctophilin 2 | TPM2 | 12011 | tropomyosin 2 (beta) |
| JUP | 6207 | junction plakoglobin | TPM3 | 12012 | tropomyosin 3 |
| KARS | 6215 | lysyl-tRNA synthetase | TRIM32 | 16380 | tripartite motif containing 32 |
| KBTBD10 | 16905 | kelch-like family member 41 | TRPV4 | 18083 | transient receptor potential cation channel, subfamily V, member 4 |
| KBTBD13 | 37227 | kelch repeat and BTB (POZ) domain containing 13 | TTBK2 | 19141 | tau tubulin kinase 2 |
| KLHL40 | 30372 | kelch-like family member 40 | TTN | 12403 | titin |
| KCNA1 | 6218 | potassium channel, voltage gated shaker related subfamily A, member 1 | TTPA | 12404 | tocopherol (alpha) transfer protein |
| KCNA5 | 6224 | potassium channel, voltage gated shaker related subfamily A, member 5 | TTR | 12405 | transthyretin |
| KCNC3 | 6235 | potassium channel, voltage gated Shaw related subfamily C, member 3 | TUBA1A | 20766 | tubulin, alpha 1a |
| KCNE1 | 6240 | potassium channel, voltage gated subfamily E regulatory beta subunit 1 | TUBB3 | 20772 | tubulin, beta 3 class III |
| KCNE2 | 6242 | potassium channel, voltage gated subfamily E regulatory beta subunit 2 | UBA1 | 12469 | ubiquitin-like modifier activating enzyme 1 |
| KCNE3 | 6243 | potassium channel, voltage gated subfamily E regulatory beta subunit 3 | UTRN | 12635 | utrophin |
| KCNH2 | 6251 | potassium channel, voltage gated eag related subfamily H, member 2 | VAPB | 12649 | VAMP (vesicle-associated membrane protein)-associated protein B and C |
| KCNJ12 | 6258 | potassium channel, inwardly rectifying subfamily J, member 12 | VCL | 12665 | vinculin |
| KCNJ18 | 39080 | potassium channel, inwardly rectifying subfamily J, member 18 | VCP | 12666 | Valosin containing protein |
| KCNJ2 | 6263 | potassium channel, inwardly rectifying subfamily J, member 2 | VMA21 | 22082 | VMA21 vacuolar H+-ATPase homolog (S. cerevisiae) |
| KCNQ1 | 6294 | potassium channel, voltage gated KQT-like subfamily Q, member 1 | VRK1 | 12718 | vaccinia related kinase 1 |
| KIAA0196 | 28984 | KIAA0196 | WNK1 | 14540 | WNK lysine deficient protein kinase 1 |
| KIF1A | 888 | kinesin family member 1A | YARS | 12840 | tyrosyl-tRNA synthetase |
| KIF1B | 16636 | kinesin family member 1B | ZFYVE26 | 20761 | zinc finger, FYVE domain containing 26 |
|  |  |  | ZFYVE27 | 26559 | zinc finger, FYVE domain containing 27 |
|  |  |  |  |  |  |

| **Table e5: Cost of diagnostic investigations in Australian dollars (AUD$)**  Items in bold are covered by the Medicare Benefits Schedule | | | | |
| --- | --- | --- | --- | --- |
| **Laboratory investigations** | | | | |
| α-glucosidase (Pompe disease) | $359.00 |  | Neuraminidase level | $646.00 |
| 7-dehydrocholesterol | $200.00 |  | Peroxisomal enzymes (DHAP-AT and DHAP-s) | $1,037.00 |
| 8-dehydrocholesterol and 8(9) cholesterol | $270.00 |  | Plasmalogens | $421.00 |
| Acid maltase deficiency | $359.00 |  | Phytanic acid | $195.00 |
| Acylcarnitine profile | $200.00 |  | **Pyruvate** | $51.95 |
| Acylcarnitine profile (fibroblasts) | $350.00 |  | Transferrin isoforms | $210.00 |
| **Amino acids (plasma)** | $98.30 |  | **Thyroid function** | $25.05 |
| **Ammonia** | $9.70 |  | **Uric acid** | $11.65 |
| Bile alcohol analysis | $482.00 |  | Very long chain fatty acids | $175.00 |
| Congenital disorder of glycosylation (type 1A and 1B) analysis | $472.00 |  | White cell enzyme analysis | $401.00 |
| Ceruloplasmin | $15.00 |  | **Acetylcholine receptor antibody** | $34.55 |
| Cholestantriol | $113.00 |  | Anti-muscle specific kinase antibody testing | $240.00 |
| **Cholesterol and triglycerides** | $11.65 |  | **TORCH screen** | $55.70 |
| CoQ10 Analysis | $85.00 |  | **Antinuclear antibody (ANA)** | $24.45 |
| **Copper** | $30.60 |  | **Double stranded DNA (dsDNA) antibody** | $26.50 |
| Carnitine palmitoyltransferase 2 enzyme analysis | $350.00 |  | **Extractable nuclear antigen (ENA) antibodies** | $17.40 |
| **Creatine kinase** | $20.05 |  | **Urine metabolic screen** | $200.00 |
| Creatine and guanidinoacetate studies (serum and urine) | $200.00 |  | Urine oligosaccharides | $195.00 |
| **Homocysteine** | $24.70 |  | Urine purine and pyrimidine analysis | $292.00 |
| **Lactate** | $9.70 |  | **CSF microscopy, culture, protein and glucose** | $72.30 |
| Methylmalonate | $200.00 |  | **CSF** **amino** **acids** | $98.30 |
| Mitochondrial enzyme analysis (muscle/liver) | $1,130.00 |  | **CSF lactate** | $24.70 |
| Mitochondrial enzyme analysis (fibroblasts) | $1,525.00 |  | CSF neurotransmitters | $250.00 |
| **Laboratory Costs** | | | | |
| Blood collection | $30.00 |  | DNA preparation for send away | $70.00 |
| Send away (Australia) (blood/urine/ fibroblasts) | $50.00 |  | Washed red cells preparation and sendaway (Adelaide) | $100.00 |
| Send away (overseas) (blood/urine/fibroblasts) | $80.00 |  | Frozen sample send away (muscle/liver) | $140.00 |
| **Laboratory Investigations – Anatomic Pathology** | | | | |
| **Histopathology for tissue specimen** | $107.05 |  | **Electron microscopy (1 specimen)** | $184.35 |
| **Histopathology for nerve biopsy** | $325.00 |  | **Electron microscopy (2+ specimens)** | $245.80 |
| Immunohistochemistry (research cost) | $550^x^ |  | Post mortem | $3100.00 |
| Western Blot (research cost) | $750^y^ |  |  |  |
| **Procedure Costs** | | | | |
| **Electrophysiological studies** | $335.95 |  | **Nerve biopsy** | $117.55 |
| **General anaesthetic for MRI** | $138.60 |  | **Liver biopsy** | $174.45 |
| **General anaesthetic for procedure** | $99.00 |  | Ward admission | $980.47 |
| **Sedation for procedure** | $99.00 |  | Day stay ward | $1,716.38 |
| **Muscle biopsy** | $149.75 |  | PICU admission for muscle biopsy | $3672.23 |
| **Skin biopsy** | $52.20 |  | **Ophthalmology review** | $192.80 |
| **Lumbar Puncture** | $75.30 |  | **Connective tissue dysplasia clinic review** | $85.55 |
| **Suction rectal biopsy** | $256.95 |  | **Metabolic clinic review** | $85.55 |
|  |  |  | **Electroencephalogram** | $800 |
| **Medical Imaging Costs** | | | | |
| **MRI brain** | $403.20 |  | **MRI muscle** | $380.80 |
| **MRI spine** | $448.00 |  | **Muscle ultrasound** | $109.10 |
| **Head ultrasound** | $54.55 |  | **Abdominal ultrasound** | $55.65 |
| **Skeletal survey** | $89.40 |  |  |  |
| **Genetic Investigations** | | | | |
| DNA extraction & storage | $100.00 |  | *LMOD3* sequencing (research cost) | $600 |
| **Chromosomal microarray** | $589.90 |  | Mitochondrial DNA point mutations | $350.00 |
| Hi resolution microarray | $589.90 |  | MELAS point mutation | $250.00 |
| **Standard karyotype** | $358.95 |  | Mitochondrial sequencing (DNA extracted from muscle) | $1,536.18 |
| Subtelomeric FISH | $650.00 |  | *MTM1* sequencing | $1,430.00 |
| 22q11 FISH | $175.00 |  | Muscle mitochondrial depletion testing | $350.00 |
| Facioscapular humeral dystrophy | $800.00 |  | *NEB* sequencing (commercial) | $6,100.00 |
| **Fragile X** | $101.30 |  | *NEB* sequencing (research cost) | $2,230.00 |
| Prader Willi syndrome (UPD) | $300.00 |  | *NEB* dHPLC | $2,977.00 |
| *SMN1* exon 7 copy number (SMA) | $300.00 |  | *NEB* haplotyping (proband) | $2,500.00 |
| Myotonic dystrophy (*DM1*) | $450.00 |  | *NEB* haplotyping (sibling) | $500.00 |
| MLPA for common microdeletion syndromes (SALSA kit) | $300.00 |  | Nemaline myopathy CNV array | $730.00 |
| *ACTA1* sequencing (research cost) | $600.00 |  | Neuromuscular gene panel (NGS) | $1,100.00 |
| *ACTG1* sequencing (research cost) | $500.00 |  | *PABN1* analysis - commercial - Queensland | $145.00 |
| α-dystroglycanopathy gene sequencing | $3,117.85 |  | *PNPLA2* sequencing | $1,150.00 |
| *BAG3* sequencing (research cost) | $500.00 |  | *RYR1* sequencing (Ion torrent) (research cost) | $2,500.00 |
| *CAPN3* sequencing (research cost) | $950.00 |  | *SEPN1* sequencing (research cost) | $1,100.00 |
| *CAV3* sequencing (research cost) | $200.00 |  | *SGCE* sequencing | $910.98 |
| *CNBP* sequencing (research cost) | $600.00 |  | *TGFBR1* and *TGFBR2* sequencing | $900.00 |
| Collagen VI IHC and sequencing Melbourne (research cost) | $3,004.69 |  | *TNNT1* sequencing (research cost) | $1200.00 |
| Confirmation Sanger sequencing and segregation studies (per variant) | $200.00 |  | *TPM2* sequencing (research cost) | $1,000.00 |
| *DNM2* sequencing (research cost) | $1,800.00 |  | *TPM3* sequencing (research cost) | $1000.00 |
| Dystrophin MLPA | $440.00 |  | *TRPV4* sequencing | $1,248.00 |
| Dystrophin sequencing | $1,400.00 |  | *UBE1* sequencing | $430.00 |
| *FHL1* sequencing (research cost) | $800.00 |  | Whole exome sequencing (proband) | $2600.00 |
| *FKRP* sequencing (research cost) | $400.00 |  | Whole exome sequencing (trio) | $7100 |
| *IGHMPB2* sequencing | $1,279.08 |  | Fibroblast cell line - retrieval | $120.00 |
| *LAMA2* sequencing | $3,621.00 |  | Fibroblast cell line- establish | $350.00 |
| *LMNA* sequencing (research cost) | $1,000.00 |  | Myoblast cell line | $400.00 |
| *MATR3* sequencing (research cost) | $1,600.00 |  |  |  |
|  |  |  |  |  |
|  |  |  |  |  |
| ^x^ Immunohistochemistry costs based on $50 per antibody including reagents and labour plus 2 hours microscopy (@$50 per hour) per six antibodies. $150 for repeat staining and microscopy per 6 antibodies.  ^y^ Western blot costs based on 8 hours labour per W.blot (@$50 per hour); $100 reagents; and repeat of abnormality.  CNV – copy number variant; CSF – cerebrospinal fluid; FISH – fluorescent in situ hybridization; IHC – immunohistochemistry; MLPA – Multiplex Ligation-dependent Probe Amplification; NGS – Next generation sequencing; SMA – spinal muscular atrophy; TORCH – Antibody screen for congenital infection; UPD – uniparental disomy | | | | |
